# Population pharmacokinetic and covariate analyses of intravenous trastuzumab (Herceptin^®^), a HER2-targeted monoclonal antibody, in patients with a variety of solid tumors

**DOI:** 10.1007/s00280-018-3728-z

**Published:** 2018-11-22

**Authors:** Angelica L. Quartino, Hanbin Li, Whitney P. Kirschbrown, Ranvir Mangat, D. Russell Wada, Amit Garg, Jin Y. Jin, Bert Lum

**Affiliations:** 1 Genentech, Inc., 1 DNA Way, Mail Stop 463A, South San Francisco, CA 94080 USA; 2 Certara, L.P., 845 Oak Grove Ave, Menlo Park, CA 94025 USA; 3 Present Address: Insight Rx, 233 Stanyan Street, San Francisco, CA 94118 USA

**Keywords:** Early breast cancer, Metastatic breast cancer, Advanced gastric cancer, Population pharmacokinetics, Trastuzumab, Herceptin

## Abstract

**Purpose:**

The aim of the study was to characterize the population pharmacokinetics (PK) of the intravenous formulation of trastuzumab, assess the impact of patient and pathological covariates on trastuzumab PK, and perform simulations to support dosing recommendations in special situations.

**Methods:**

Serum trastuzumab concentrations were obtained from 1582 patients with metastatic breast cancer (MBC), early breast cancer (EBC), advanced gastric cancer (AGC), or other tumor types/healthy volunteers in 18 phase I, II, and III trials and analyzed by nonlinear mixed-effects modeling.

**Results:**

A two-compartment model with parallel linear and nonlinear elimination best described the data. During treatment, linear clearance (CL) dominated, resulting in a total CL of 0.173–0.337 L/day, which is similar to other IgG1 monoclonal antibodies. Covariates influencing CL were baseline body weight, aspartate aminotransferase, albumin, gastric cancer, and the presence of liver metastases. MBC and EBC had similar PK parameters, while CL was higher in AGC. Simulations indicated that at least 95% of patients with BC reach concentrations < 1 µg/mL (~ 97% washout) by 7 months. A dose delay in BC or AGC patients of > 1 week would take approximately 6 weeks to get back within steady-state exposure range.

**Conclusions:**

Trastuzumab PK for the intravenous formulation was well-described across cancer types, disease status, and regimens. No dose adjustment is required for any of the identified patient covariates. A 7-month serum washout period for trastuzumab is recommended. A reloading dose is required if a maintenance dose is missed by > 1 week.

**Electronic supplementary material:**

The online version of this article (10.1007/s00280-018-3728-z) contains supplementary material, which is available to authorized users.

## Introduction

Trastuzumab (Herceptin^®^; Genentech Inc., South San Francisco, CA, USA), a humanized immunoglobulin G1 (IgG1) monoclonal antibody (mAb), binds selectively to the extracellular domain of HER2 with high affinity, resulting in inhibition of downstream signaling pathways, cell cycle arrest, and reduction in angiogenesis [1, 2]. It was developed as an intravenous (IV) formulation as a targeted therapy for the management of HER2-positive breast cancer (BC) and became the first FDA-approved biologic for the treatment of solid tumors in 1998 (for HER2-positive metastatic BC [MBC]). Trastuzumab was tested clinically in several phase I–III trials in patients with a variety of solid tumors and is currently approved as monotherapy or in combination with chemotherapy for patients with HER2-positive early BC (EBC), MBC, and advanced gastric cancer (AGC). Dosing of the IV formulation includes an 8 mg/kg loading dose followed by 6 mg/kg maintenance doses every 3 weeks (q3w) for EBC/MBC and AGC, or 4 mg/kg loading dose followed by 2 mg/kg weekly (qw) maintenance doses for BC.

Trastuzumab population pharmacokinetics (PopPK) has been characterized in MBC [3], EBC [4], and AGC [5]. Depending on the study design, dosing regimen, and PK sampling schedule, both linear and nonlinear trastuzumab elimination have been observed; the latter presumably reflecting target-mediated drug disposition [6]. These models generally report a total clearance (CL) at steady state of approximately 0.24 L/h and a terminal elimination half-life between 25 and 30 days following the q3w IV regimen in BC.

The first PopPK model described for IV trastuzumab was a linear two-compartment model based on PK data from 476 patients with MBC in phase I–III studies using qw dosing [3]. Body weight (WT), HER2 extracellular domain plasma levels, and the number of metastatic sites were significant covariates influencing linear CL and/or volume of distribution. Later, in a HER2-positive EBC population, the model with nonlinear elimination was found to best describe trastuzumab PK [4]. In this analysis, WT and alanine transaminase were identified as significant covariates influencing CL and central volume (*V*_c_). The nonlinear model was also used to best describe the PK in the AGC population [5]. WT, prior gastrectomy, and serum albumin (ALBU) had significant influence on CL, and concentrations were significantly lower than those observed in BC.

Subtle but important differences in patient data (e.g., the presence/absence of Michaelis–Menten kinetics), significant covariates, and dosing regimens included in each of the previous PopPK models make it difficult to provide useful information for healthcare providers to manage patients receiving trastuzumab for various clinical scenarios (e.g., missed doses, washout period for pregnancy).

Taking into account the study-specific differences in disease status, dose regimens, and demographic variables and serum trastuzumab concentration–time points, data were compiled from 18 phase I–III trials, to allow building a comprehensive PopPK model for IV trastuzumab.

The key objectives of this analysis were to:

1. characterize the PopPK of IV trastuzumab across a variety of solid tumors, disease statuses, and dosing regimens;
2. assess the impact of patient characteristics and pathophysiological covariates on trastuzumab PK;
3. conduct simulations using the PopPK model to provide dosing recommendations for drug washout (treatment-free) periods for situations such as use of anthracycline-based therapy, pregnancy, or breastfeeding, and also to provide recommendations for modification of dose regimen in cases of missed doses to minimize PK underexposure and quickly re-establish trastuzumab steady-state concentrations.

## Patients and methods

### Studies and patients

Trastuzumab serum PK samples collected across 18 phase I–III trials were included. All studies used the innovator trastuzumab product Herceptin^®^; no patients received biosimilars. Details of the studies are summarized in Online Resource 1.

All were conducted in accordance with the Declaration of Helsinki, with all protocols and amendments approved by independent ethics committees. All participants provided written informed consent.

### Serum trastuzumab concentration assays

Trastuzumab serum concentrations were determined using a validated enzyme-linked immunosorbent assay [7] with a lower limit of quantification of 0.156 µg/mL.

### PK data handling

Patients were defined as evaluable for PK analysis if they had ≥ 1 adequately documented trastuzumab dose and ≥ 1 corresponding concentration sample. If the dose date was missing, or if the PK sample was below the limit of quantification, the sample was omitted (*n* = 316). In total, 1588 patients and 27,370 samples were considered valid.

Handling of outliers is discussed in Online Resources 2–3.

### Population PK analysis

PopPK methods were based on FDA [8] and EMA [9] regulatory guidance. Trastuzumab PK data were analyzed by nonlinear mixed-effects modeling with NONMEM Version 7.2 (ICON Development Solutions, Ellicott City, MD, USA) with the subroutine ADVAN6 using the first-order conditional estimation method with interaction. Model selection was based on the likelihood ratio test (*p* < 0.001). In the case that the $COV step was not completed, Monte Carlo importance sampling (IMP) and bootstrapping were used to obtain the standard errors for the final model. Perl-speaks-NONMEM (PsN, version 3.2, http://psn.sourceforge.net/) was used to aid model development; S-Plus software (Version 8.1, TIBCO Software Inc., Palo Alto, CA), and the R Software package (Version 3.0, http://www.r-project.org/) were used for data assembly, exploratory data analysis, model diagnosis, and model simulation.

Model evaluation is discussed in Online Resource 4.

### Covariate analysis

Potential PK covariates were selected based on biologic and clinical relevance, and previous experience with trastuzumab or other IgG1 mAbs. The following covariates were considered in the model building: age, sex, race (Asian/non-Asian), baseline WT, primary tumor type (MBC/EBC/AGC/other tumor types), baseline Eastern Cooperative Oncology Group (ECOG) performance status (0/1 versus ≥ 2), baseline number of metastatic sites (0–3 versus ≥ 4), baseline presence of liver metastases (LMET, yes/no), baseline HER2 immunohistochemistry overexpression, regimen (qw/q3w), single agent versus combination with chemotherapy, and baseline levels of aspartate aminotransferase (SGOT), alanine aminotransferase (SGPT), total serum bilirubin (TBIL), ALBU, creatinine clearance (CrCL), and alkaline phosphatase (ALK). When covariate data were missing for ≤ 10% of patients, continuous covariates were imputed as the population median, and categorical covariates were grouped with the predominant category. When covariate data were missing for > 10% of patients, no imputation occurred, and an exploratory analysis was conducted in patients with available covariate information. First, graphs of Empirical Bayes (“post hoc”) Estimates (EBEs) of the PK parameters versus the covariates of interest were examined. Second, the covariate was tested in NONMEM for significance (*p* < 0.001) in a data subset.

The three-step forward addition and backward elimination approach that was used to identify covariates is discussed in Online Resource 5.

The effect of covariates on model-predicted exposures in a typical patient was assessed by changing one covariate at a time and fixing all other covariates of the typical population. If the covariate examined had an impact on multiple PK parameters [i.e., *V*_c_, volume of distribution (peripheral compartment; *V*_p_) and CL], its impact was applied to all PK parameters simultaneously.

### Simulations for washout and missed doses

Simulations using the final PopPK trastuzumab model were used to: (1) determine the length of a washout period following different IV dosing regimens and intervals, and (2) describe concentrations where a dose was missed by a range of days.

The time needed for trastuzumab washout was based on Monte Carlo simulations of serum concentrations following 36 and 12 treatment cycles (steady state) for the 4 mg/kg loading dose + 2 mg/kg qw and 8 mg/kg loading dose + 6 mg/kg q3w dosing regimens, respectively. Time to steady state (90%) was 12 weeks for both regimens, but simulations to 36 weeks were performed to capture 100% steady state. The washout time period was evaluated by the time required for the trastuzumab concentration to reach < 1 µg/mL [approximately 3% of steady-state *C*_min_ concentrations (*C*_min,ss_) or 97% washout] for 95% of the virtual patients. This is consistent with the PK principle of the percentage of drug washout from systemic circulation following five elimination half-lives.

To describe trastuzumab concentrations following situations of a missed dose, simulations for a typical patient with BC and a typical patient with AGC (WT = 66 kg; SGOT = 24 IU/L; ALBU = 4 g/dL, and without LMET at baseline) using the PopPK model were performed. Trastuzumab concentrations were simulated for clinical scenarios following a missed dose of 1 or 2 weeks’ duration (dose delays). Simulations were conducted such that the subsequent clinical management following the dose delay was either readministration of a loading dose or continuation with the maintenance dose every 7 or 21 days, for the qw or q3w regimen, respectively. These scenarios were compared to the situation where there was no missed dosing. The recommendation for the readministration of a loading dose was evaluated based on the time required for *C*_min,ss_, after missing a dose, to return within 15% of the *C*_min,ss_ level given no missed dose.

## Results

### Patient population

The final PK model dataset contained 1582 patients and 26,040 trastuzumab serum PK samples across 18 studies. A final sensitivity analysis was performed where all data including outliers were used, which resulted in similar parameter estimates.

Patient demographic characteristics are summarized in Online Resource 6. Most patients had MBC (810/1582), followed by EBC (391), AGC (274), non-small cell lung cancer (NSCLC) or other tumor types (107), and healthy volunteers (HV; 6 subjects). Overall, 82.7% were female and 17.3% were male. Median age and WT was 53 years and 66 kg, respectively. Most (1324/1588) patients were non-Asian (83.4%) and 94.5% had an ECOG performance status of 0 or 1. Among patients with AGC, 12.6% had prior gastrectomy.

Patients received trastuzumab as single agent (*n* = 1188) or in combination with chemotherapy (anthracyclines, docetaxel, paclitaxel, cisplatin, or other chemotherapy) on either a q3w (*n* = 917) or qw (*n* = 643) schedule or as a single dose (*n* = 28).

### Trastuzumab PK

The final PK model was a two-compartment model with parallel linear and nonlinear (Michaelis–Menten) elimination from the central compartment. PK parameter estimates for trastuzumab are presented in Table 1. These estimates were well defined (relative standard error < 10%) as assessed by the IMP method and a stratified nonparametric bootstrap procedure. Model building started with a two-compartment model with linear CL. The addition of the nonlinear CL component to the model decreased the objective function value by 871 points (*p* < 0.001). The nonlinear elimination was also supported by graphical inspection of the concentration data, in particular at the lower concentrations following the first dose (data not shown).

**Table 1:** Final population pharmacokinetic parameters for IV trastuzumab

Name	Parameter description	Estimate (% RSE)^a^	Bootstrap mean (% RSE)^b^
*θ*_1_	Linear elimination clearance, CL (L/day)	0.127 (2.36)	0.126 (2.66)
*θ*_8_	Linear CL for other tumor types	0.148 (5.81)	0.147 (4.55)
*θ*_9_	Linear CL for AGC patients	0.176 (4.19)	0.175 (4.21)
*θ*_2_	Volume of distribution, central compartment for non-AGC patients, *V*_c_ (L)	2.62 (0.79)	2.62 (0.629)
*θ*_13_	Volume of distribution, central compartment for AGC patients, *V*_c_ (L)	3.63 (1.94)	3.63 (1.92)
*θ*_3_	Distribution clearance, *Q* (L/day)	0.544 (3.38)	0.543 (3.81)
*θ*_4_	Volume of distribution, peripheral compartment, *V*_p_ (L)	2.97 (1.81)	2.97 (1.78)
*θ*_5_	*V*_max_ (mg/day)	8.81 (1.44)	8.86 (4.09)
*θ*_6_	*K*_m_ (mg/L)	8.92 (8.61)	8.97 (14.5)
*θ*_7_	Influence of WT on linear CL	0.967 (7.19)	0.973 (6.34)
*θ*_10_	Influence of SGOT on linear CL	0.205 (16.6)	0.211 (15.5)
*θ*_11_	Influence of ALBU on linear CL	− 0.998 (12.2)	− 1 (12.2)
*θ*_12_	Influence of LMET on linear CL	0.152 (21.4)	0.148 (20.5)
*ω*_CL_^c^	IIV of CL (%)	40.1 (6.71)	40.1 (7.49)
${\omega _{{{V_{\text{c}}}}}}^{{\text{c}}}$	IIV of *V*_c_ (%)	24.6 (4.98)	24.5 (4.93)
${\omega _{{V_{\text{p}}}}}$	IIV of *V*_p_ (%)	49.5 (9.39)	49.6 (7.51)
${\omega _{{K_{\text{m}}}}}$	IIV of *K*_m_ (%)	139 (20.3)	141 (11.9)
σ_1_^d^	Proportional variability (%)	19.7 (1.35)	19.7 (1.32)
σ_2_^d^	Additive variability (µg/mL)	1.38 (31.8)	1.33 (28.2)
Shrinkage (%)			
$\omega _{{{\text{CL}}}}^{{}}$		14.7	
$\omega _{{{V_{\text{c}}}}}^{{}}$		13.0	
${\omega _{{V_{\text{p}}}}}$		22.9	
${\omega _{{K_{\text{m}}}}}$		44.0	
σ_1,_ σ_2_		7.0	

*FOCEI* first-order conditional estimation method with interaction, *IMP* importance sampling, *IIV* inter-individual variability, *NSIG* number of significant digits, *RSE* relative standard error

^a^Model estimate using FOCEI method in NONMEM, with NSIG = 3. SE of the model estimates were obtained from IMP method in NONMEM

^b^Bootstrap results were from all 200 runs (169 runs successful and 31 runs with minimization terminated, results are similar regardless whether those terminated runs are included)

^c^Off-diagonal covariance term Ω_CL,Vc_ = 0.0230

^d^RSE for residual variability terms (*σ*_1_, *σ*_2_) is relative to the estimated variance (*σ*_1_^2^, *σ*_2_^2^)

The between-subject variability was modest except for *K*_m_ (the concentration at which the nonlinear CL rate is half of the maximum rate of nonlinear CL), which was high (139%) and was likely due to there being limited data available at low concentrations around *K*_m_ (8.92 µg/mL) for most patients.

Goodness-of-fit plots showed good agreement between predicted and observed concentrations, with no bias in residuals over time and across predicted concentration values (Online Resource 7). Inter-individual variability for each PK parameter was normally distributed around zero, which is consistent with the model assumption on inter-individual variability.

**Fig. 1:**
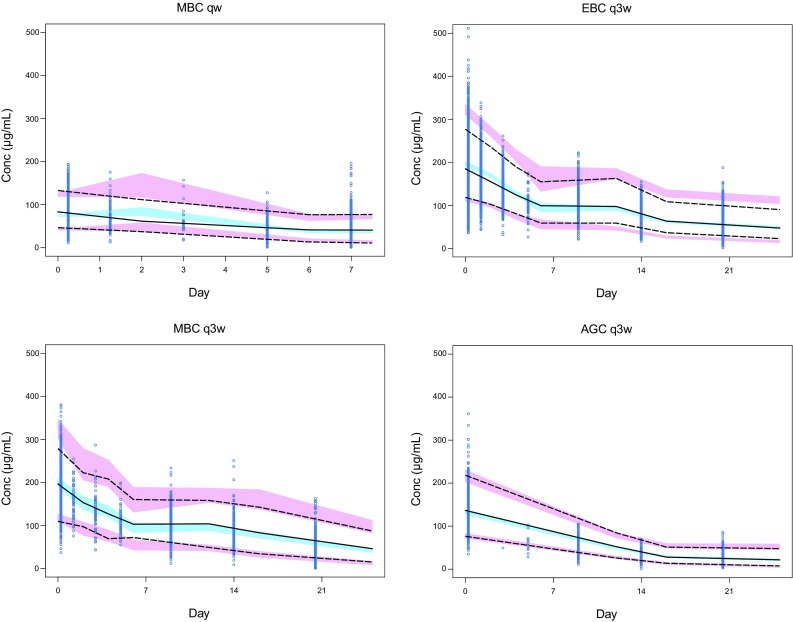
Visual predictive checks of observed and model-predicted trastuzumab concentrations for patients with MBC, EBC, and AGC. Created using S-Plus Software package (Version 8.2, SolutionMetrics, Sydney, NSW, Australia). Circles are observed trastuzumab serum concentrations; solid black lines represent the median observed value; and dashed lines represent 5 and 95% prediction intervals of the observed values. Blue shaded areas represent the 5 and 95% of the median predicted values; and red shaded areas represent the spread (5th and 95th percentile) of the predicted concentrations. *AGC* advanced gastric cancer, *EBC* early breast cancer, *MBC* metastatic breast cancer, *qw* weekly, *q3w* every 3 weeks

Visual predictive checks (VPCs; Fig. 1), stratified by primary tumor type and dose regimen, were used to evaluate the capability of the final model to reproduce the observed data. Overall, the VPC plots show that the median, fifth percentile, and ninety-fifth percentile of the PK simulated concentrations match the observed values, suggesting that the model predicts the observed concentrations reasonably well. Numerical predictive check results suggest that the final model adequately describes the distribution of trastuzumab PK concentrations, in particular the low concentration values (95%tile 3.7%, 75%tile 23.1%, 50%tile 51.6%, 50%tile 48.4%, 25%tile 20.7%, 5%tile 4.1%).

The shrinkage rates of CL, *V*_c_, and *V*_p_ were 14.7, 13 and 22.9%, respectively. *K*_m_ had greater shrinkage of 44%.

### Identification of impactful patient and pathophysiological factors which influence trastuzumab PK

WT was first added to the base model on linear CL, *V*_c_, and *V*_p_ because it was shown to have a significant impact on the PK of IV trastuzumab regimen in previous analyses, before screening other covariates. The second stepwise forward addition step identified tumor type (grouped by MBC versus EBC/HV versus AGC versus others) on CL and* V*_c_, and ALBU and SGOT on CL as the most important factors, which were added to the base model. In the third univariate-screening step, ALK, ECOG (missing, 0, 1 versus 2 or more), LMET on CL, sex, SGOT, SGPT, number of metastatic sites (0–3 versus 4 or more) on *V*_c_, and primary tumor type on *K*_m_ were found to be significant (*p* < 0.005).

Covariates were then excluded from the full model using a three-step backward elimination process. In the first elimination step, the effects of ECOG status and ALK on CL, SGPT on *V*_c_, and primary tumor type on *K*_m_ were excluded from the model (*p* < 0.001). In the next step, the four primary tumor type groups (MBC, EBC/HV, AGC, and others) were compared to the reference MBC population. No formal evaluation was possible for HV versus EBC because the dataset included only six HVs. BC patients, i.e., MBC and EBC/HV, had similar CL; AGC and other tumor types had higher CL compared with MBC (*p* < 0.001). The *V*_c_ values were significantly different among the four primary tumor type groups. The *K*_m_ value was similar in all patient populations apart from AGC, which had significantly lower *K*_m_.

The final statistical model was used to evaluate the impact of the covariates on steady-state trastuzumab exposure [*C*_min,ss_, maximum serum concentration (*C*_max_)_,ss_, and area under the curve (AUC)_ss_] by simulating a typical MBC patient (WT = 66 kg, SGOT = 24 IU/L, ALBU = 4.0 g/dL, and without LMET) dosed with an 8 mg/kg loading dose followed by the 6 mg/kg q3w maintenance regimen. The covariates in the final model that met the clinical relevance criterion (15% impact on steady-state exposure and difference from typical MBC patients for categorical variables) were WT, ALBU, SGOT, primary tumor types (MBC/EBC/HV versus AGC versus NSCLC and others) and LMET on CL, and AGC on *V*_c_. None of the statistically significant covariates met the clinical relevance criteria on *V*_p_ or *K*_m_.

The final covariate relationships are given as

$$\begin{aligned} {\text{C}}{{\text{L}}_i}\;= & \;\left[ {{\theta _1} \cdot ({\text{TTYP}}{{\text{E}}_i}\;==\;{\text{MBC~or~EBC~or~HV)}}~+{\theta _9} \cdot ({\text{TTYP}}{{\text{E}}_i}=={\text{AGC)}}} \right.~ \\ & \;\left. {+\;{\theta _8} \cdot \left( {{\text{TTYP}}{{\text{E}}_i}=={\text{Others}}} \right)} \right] \cdot {\left( {\frac{{{\text{W}}{{\text{t}}_i}}}{{66}}} \right)^{{\theta _7}}} \cdot {\left( {\frac{{{\text{SGO}}{{\text{T}}_i}}}{{24}}} \right)^{{\theta _{1102}}}} \cdot {\left( {\frac{{{\text{ALB}}{{\text{U}}_i}}}{4}} \right)^{{\theta _{11}}}} \cdot {{\text{e}}^{\left( {{\theta _{12}} \cdot \left[ {{\text{LME}}{{\text{T}}_i}=Y} \right]} \right)}} \cdot {{\text{e}}^{{\eta _{{\text{CL}}}}}} \\ V{{\text{c}}_i}{\text{ }}= & {\text{ }}[{\theta _2} \cdot ({\text{TTYP}}{{\text{E}}_i}=={\text{Non-AGC}})~+{\theta _{13}} \cdot ({\text{TTYP}}{{\text{E}}_i}=={\text{AGC}})~] \cdot {{\text{e}}^{{\eta _{{\text{Vc}}}}}} \\ \end{aligned}$$

where *θ* population parameter value, TTYPE tumor type, and *i* index for subject.

### Assessment of the impact of identified covariates on PK exposure

**Fig. 2:**
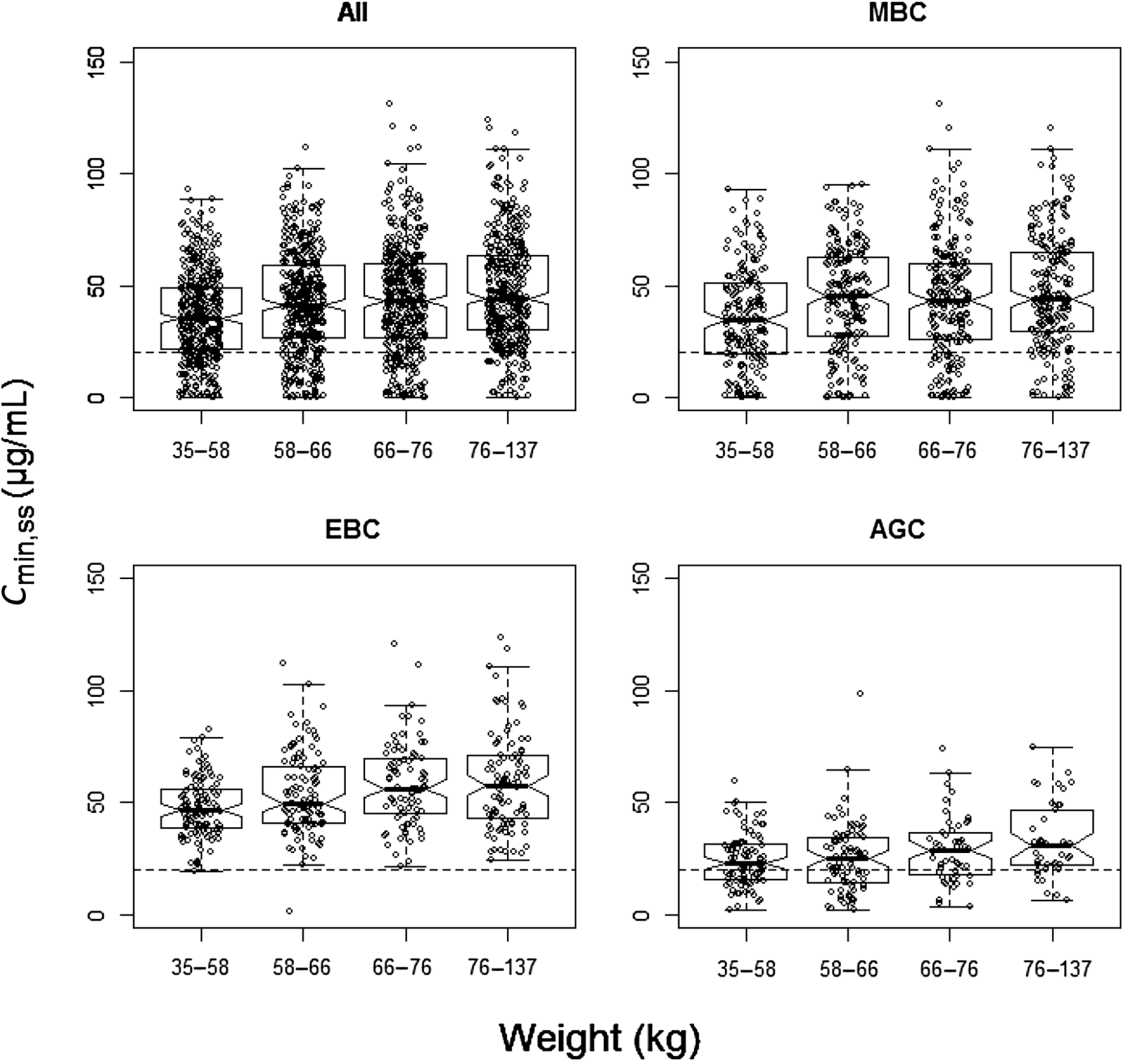
Impact of baseline body weight on model-predicted steady-state *C*_min,ss_ stratified by primary tumor type for a q3w regimen of an 8 mg/kg loading dose followed by 6 mg/kg q3w. Created using R Software package (version 3.0, http://www.r-project.org/). *AGC* advanced gastric cancer, *C*_*min,ss*_ minimum steady-state serum concentration, *EBC* early breast cancer, *MBC* metastatic breast cancer, *q3w* every 3 weeks

*WT* The covariate with the largest influence was WT. A plot of weight normalized linear clearance suggested CL appeared to be proportional to baseline WT (data not shown). When compared with the value of linear CL for a patient weighing 66 kg, the linear CL decreased 27% and increased 43% for patients weighing 46 kg and 98 kg, respectively. Patients with a higher baseline WT receive a higher absolute dose because the IV regimen was weight-adjusted (mg/kg). This weight-adjusted dosing slightly overcompensates for WT and results in a greater *C*_min,ss_ for higher WT patients as shown in Fig. 2. This overcompensation is also observed irrespective of the primary tumor type (Fig. 2).

*Serum chemistries* ALBU and SGOT: Trastuzumab PK exposure decreases with lower ALBU or higher SGOT levels. The decrease of *C*_min,ss_ is more evident when SGOT is above 36 IU/L, the upper limit of normal range for women. Online Resources 8 and 9 show that similar trends for these decreases in trastuzumab PK exposure were observed across the different primary tumor types.

**Fig. 3:**
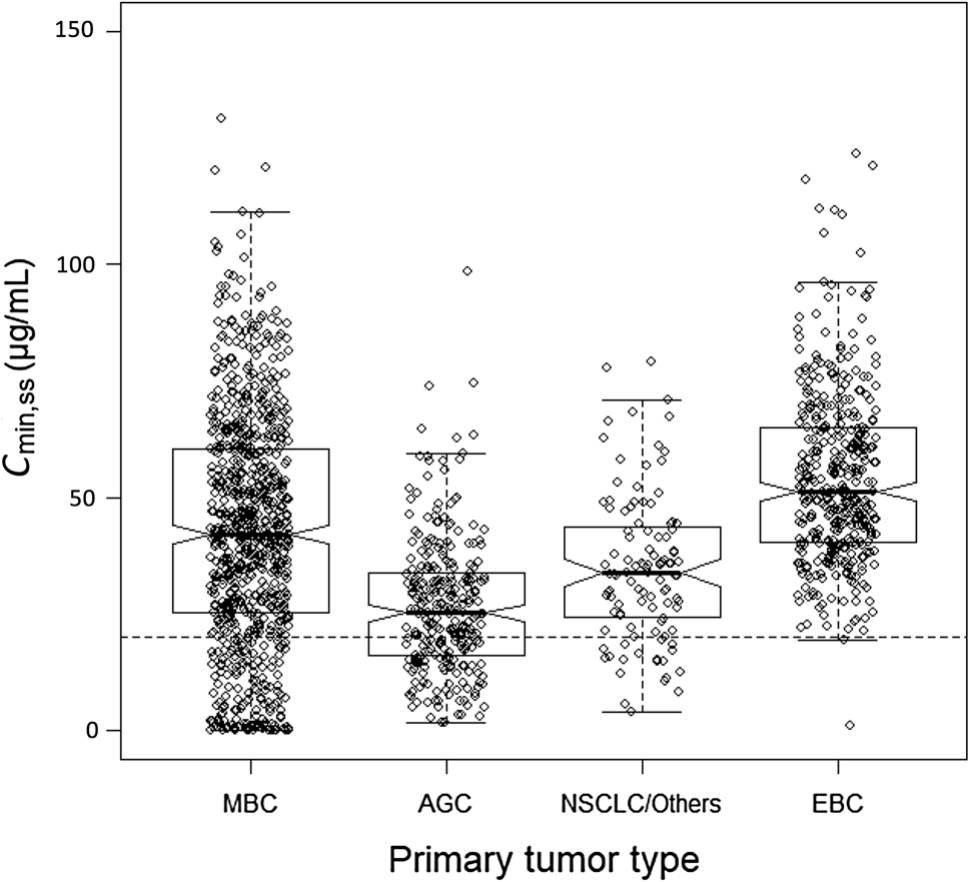
Impact of primary tumor type on model-predicted steady-state *C*_min,ss_ for an 8 mg/kg loading dose followed by 6 mg/kg q3w. Created using R Software package (Version 3.0, http://www.r-project.org/). *AGC* advanced gastric cancer, *C*_*min,ss*_ minimum steady-state serum concentration, *EBC* early breast cancer, *MBC* metastatic breast cancer, *NSCLC* non-small cell lung cancer, *q3w* every 3 weeks

*Tumor type* MBC and EBC patient populations had similar trastuzumab PK exposure, while patients with AGC had lower exposure than the other tumor types. The influence of tumor type on* C*_min,ss_ for an 8 mg/kg loading dose followed by 6 mg/kg q3w is shown in Fig. 3. Sensitivity plots indicate the difference in CL between the patients with MBC and AGC translated into a 30.5% lower* C*_min,ss_ in the AGC population using model-predicted exposure measures for an 8 mg/kg loading dose followed by 6 mg/kg q3w in a typical patient (Online Resource 10). The difference between other tumor types and MBC was modest; however, these results were interpreted cautiously, because the patients with other tumor types (mainly NSCLC, but also prostate, ovarian, etc.) were a heterogeneous group with limited patient numbers. The MBC population had a wider range in steady-state exposure compared to the EBC population, which is explained by the greater variability in ALBU, SGOT, and LMET. Almost all EBC patients had *C*_min,ss_ values above 20 µg/mL, whereas approximately 20% of patients with MBC had values below 20 µg/mL. Patients with MBC had higher SGOT and lower ALBU values than patients with EBC, and SGOT was especially higher in patients with predicted *C*_min,ss_ values below 20 µg/mL. Patients with above-normal SGOT values also tended to have low ALBU.

*Shed-antigen ECD-HER2* Shed-antigen ECD-HER2 (SHED) measurements were available in 774/1588 (47%) patients. Linear CL increased in patients with higher SHED, while *K*_m_ decreased in patients with higher SHED, indicating a possible increase of linear and nonlinear CL with higher SHED levels and, therefore, decreased exposures. As an exploratory analysis using the final statistical model, the impact of SHED on *K*_m_ was found to be significant. The coefficient of SHED on *K*_m_ was estimated as − 0.771. Simulated *C*_min,ss_ versus SHED level is illustrated in Online Resource 11.

### Simulations for dosing regimens

**Fig. 4:**
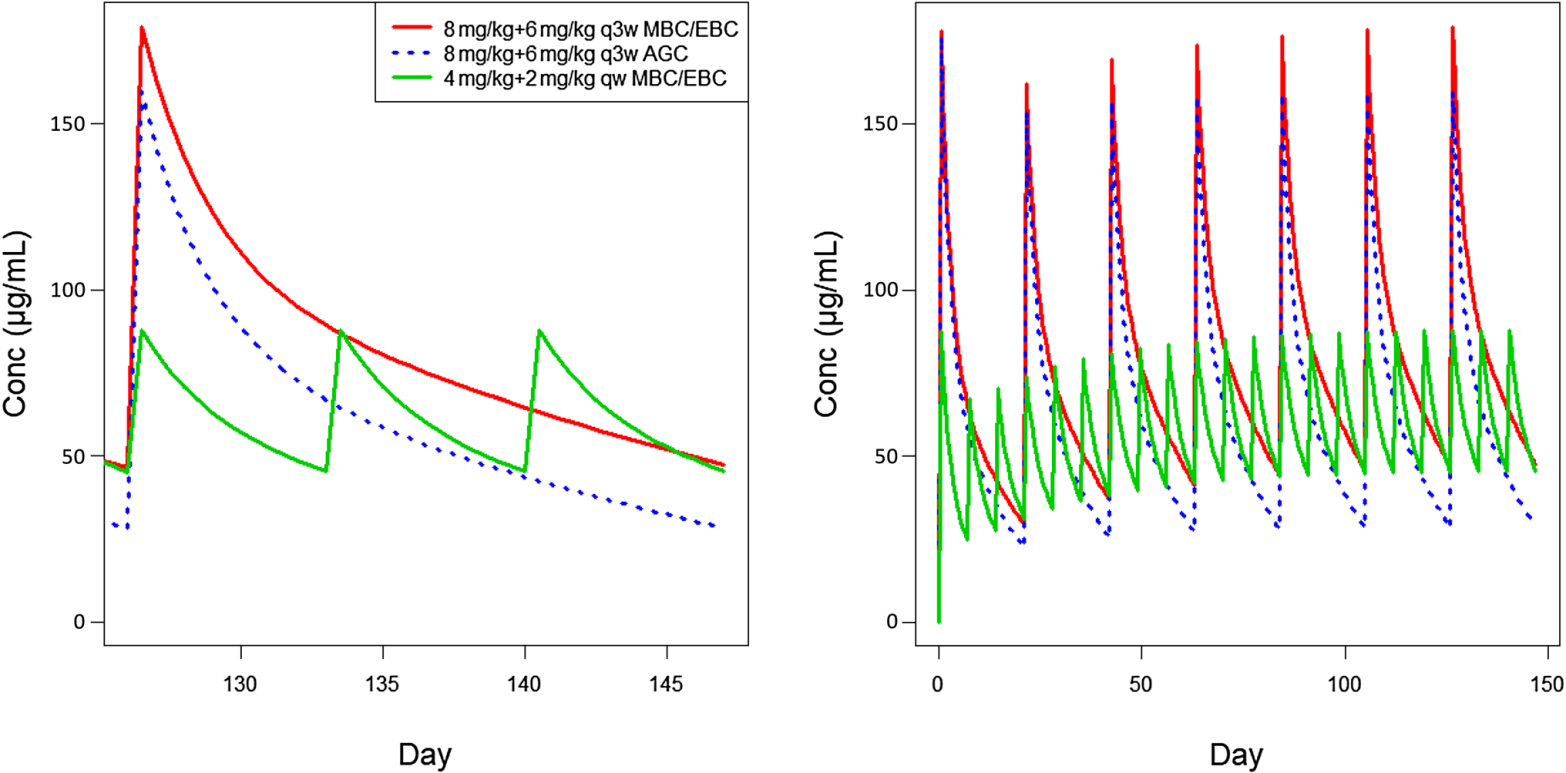
Model-predicted concentration–time profiles for the IV regimens in patients with breast cancer and AGC. Created using R Software package (Version 3.0, http://www.r-project.org/). *AGC* advanced gastric cancer, *EBC* early breast cancer, *IV* intravenous, *MBC* metastatic breast cancer

No effect of dose regimen on PK of trastuzumab was detected (*p* < 0.001). Typical predicted trastuzumab concentration–time profiles (Fig. 4) were compared for 8 mg/kg loading dose followed by 6 mg/kg q3w in BC or AGC, or 4 mg/kg loading dose followed by 2 mg/kg IV qw in BC. Treatment using the approved q3w regimen results in concentrations where linear CL dominates, with a total CL ranging from 0.173 to 0.283 and 0.189 to 0.337 L/day, resulting in 12 and 9 weeks to reach steady state in BC and AGC, respectively (Table 2, Online Resource 12). In patients with BC, the q3w regimen had lower *C*_min,ss_ and slightly greater AUC_ss_ compared to the qw regimen. *C*_min,ss_ and AUC_ss_ median values were 33% and 27% lower for the patients with AGC compared to patients with BC maintained with similar q3w regimen (Table 2).

**Table 2:** Model-predicted steady-state PK exposures (median and 95% CI) for IV regimens in patients with breast cancer and AGC

Regimen	Primary tumor type	*N*	*C*_min,ss_ (µg/mL)	*C*_max,ss_ (µg/mL)	AUC_ss_ (µg day/mL)	Time to steady state (week)	Total CL range from *C*_max,ss_ to *C*_min,ss_ (L/day)
8 mg/kg + 6 mg/kg q3w	MBC/EBC	1195	45.8 (4.56–85.5)	182 (126–260)	1790 (727–2760)	12	0.173–0.283
	AGC	274	25.2 (6.37–52.7)	119 (77.9–173)	1120 (596–1840)	9	0.189–0.337
4 mg/kg + 2 mg/kg qw	MBC/EBC	1195	65.6 (15.4–108)	109 (59.2–163)	1760 (685–2720)	12	0.201–0.244

*AGC* advanced gastric cancer, *AUC*_*ss*_ area under the concentration–time curve at steady state, *CL* linear elimination clearance, *C*_*max,ss*_ maximum steady-state serum concentration, *C*_*min,ss*_ minimum steady-state serum concentration, *EBC* early breast cancer, *MBC* metastatic breast cancer, *PK* pharmacokinetic

### Simulations for trastuzumab washout and missed doses

*Simulations of trastuzumab washout* The trastuzumab washout time period for patients with BC following 12 cycles of treatment with the 8 mg/kg loading dose + 6 mg/kg q3w dosing regimen was determined using clinical trial simulation methods. This demonstrated the nonlinear nature of trastuzumab decay with the characteristic faster elimination at the lower concentrations. The simulations show that by slightly over 6 months (188 days) following the last dose, ≥ 95% of patients have reached concentrations that are < 1 µg/mL (or approximately 3% of the *C*_min,ss_ of the typical patient with BC [*C*_min,ss_ = 47.8 µg/mL at cycle 13 pre-dose]; i.e., > 97% of the drug is washed out). A similar washout time period was obtained for the qw regimen.

*Simulations of missed doses* Simulations for patients administered the 4 mg/kg loading dose + 2 mg/kg maintenance dose weekly or the 8 mg/kg loading dose + 6 mg/kg maintenance dose every 3-week regimen to a typical patient with BC (WT = 66 kg; SGOT = 24 IU/L; ALBU = 4 g/dL, without LMET at baseline) using the final PopPK model, indicated that 90% of *C*_min,ss_ is reached by approximately 12 weeks (Online Resources 13 and 14).

In the clinical scenario, where a dose was delayed by 1 week and dosing was restarted with the 2 mg/kg (qw) or 6 mg/kg maintenance dose (q3w), there is a decline in predicted *C*_min,ss_ levels. However, concentrations return to within 15% of *C*_min,ss_ within a 3-week time period (Online Resources 15 and 16). When a dose was delayed by 2 weeks and dosing was restarted with the 2 mg/kg (qw) or 6 mg/kg maintenance dose (q3w), there is a substantial drop in trough concentration levels that do not return to within 15% of *C*_min,ss_ for approximately 6 weeks (Online Resources 17 and 18). However, in the scenario where the dose has been delayed by 2 weeks and dosing is restarted with a loading dose of 4 mg/kg (qw) or 8 mg/kg (q3w) followed by the maintenance doses of 2 mg/kg on a weekly schedule or 6 mg/kg every 3-week schedule, *C*_min_ levels return to within 15% of the *C*_min,ss_ during the first dosing interval following the administration of the reloading dose (Online Resources 19 and 20).

As expected, serum trastuzumab concentrations for the typical patient with AGC were lower than those for a typical patient with BC. However, the simulations for a missed dose in AGC were similar to those observed for the typical patient with BC. The simulations resulted in the same time for *C*_min_ levels to return to within 15% of the *C*_min,ss_, and similar requirements of a reloading or maintenance dose to achieve *C*_min,ss_ were observed in AGC compared with BC.

## Discussion

Demographic variables and serum trastuzumab concentration–time data were collected in 18 phase I, II, and III trials to build a comprehensive PopPK model for the IV trastuzumab formulation. In this analysis, a two-compartment PK model with parallel linear and nonlinear pathways (Michaelis–Menten) from the central compartment best described the concentration–time data with the final population PK model parameters listed in Table 1. Treatment using the approved q3w regimen results in concentrations where linear clearance dominates, with total CL ranging from 0.173 to 0.283 and 0.189–0.337 L/day, in BC and AGC, respectively (Table 2). These values are similar to other IgG1 mAbs. Nonlinear PK is expected at lower concentrations due to target-mediated drug disposition (TMDD); however, this was not quantified in the first PopPK model in MBC patients as a likely result of the studies in the model, which did not have low trastuzumab concentrations due to qw dosing and insufficient washout periods [3].

Target-mediated CL for trastuzumab was suggested previously by Bernadou et al. [10]. In a PopPK analysis in HER2-positive non-metastatic breast cancer, an increase in trastuzumab CL was observed with increased tumor burden/size when only a linear elimination pathway was considered. TMDD was also assumed following the administration of low doses of the antibody-drug conjugate ado-trastuzumab–emtansine (T-DM1); however, limited data at these low dose levels did not allow for estimation of model parameters associated with nonlinear elimination with good precision [11, 12]. Furthermore, as reported by Ternant et al. [13], linear PK is often interpreted as saturation of the target antigen by mAb. However, apparent linear PK does not necessarily imply an actual saturation of antigenic target by mAb; notably, where an antibody is in stoichiometric default compared with an antigen. As parallel linear and nonlinear elimination pathways are typical of mAbs with disposition that is affected by binding to a TMDD [14–17], it was appropriate to account for both pathways in the updated, robust PopPK model presented in this manuscript.

The analysis for demographic and pathophysiological covariates which influence trastuzumab PK identified baseline WT, SGOT, ALBU, primary tumor type and LMET as significant covariates on linear CL and AGC on *V*_c_. These factors in isolation altered *C*_min,ss_ by up to ± 50%. The effects on AUC_ss_ and *C*_max,ss_ were minimal (± 30%). Among the covariates tested, variability in WT was the biggest contributor to variability in trastuzumab CL based on the percentage change from a typical patient; − 48 to 59% at the 5th and 95th WT percentiles, respectively (isolated impact of covariates on PK is shown in tornado plots in Online Resource 10). This is typical for many therapeutic mAbs [18–20], and was reported previously for trastuzumab [3–5]. This likely reflects the catabolic elimination pathway of mAbs occurring in all tissues throughout the body similar to endogenous IgG. This hypothesis is supported by the fact that weight-normalized CL was constant across patients at baseline (theta estimate of WT on CL = 0.97).

The trend of increasing *C*_min,ss_ with increasing WT may be explained as follows: dosing on a mg/kg basis overcompensates for WT because the linear CL is dose proportional, but nonlinear CL is not. Nonlinear CL is approximately half the total CL at *C*_min,ss_. Accordingly, greater exposures are expected in patients with heavier WT, when dosing on a mg/kg basis. There was a visual trend for a *C*_min,ss_ increase with WT, but this was not clinically relevant.

While there appears to be a consensus regarding the contribution of WT to variability in mAb PK, there are other important covariates that can potentially contribute to the observed inter-subject variability and should be explored during clinical development. These may include factors such as age, sex, ethnicity, disease status, immune status, co-morbidities, endogenous IgG, concomitant medications, genetic polymorphisms (i.e., FcRn or FcγR) and differences in target antigen concentration [21]. ALBU was inversely correlated to CL as seen in other trastuzumab PopPK analyses and with other mAbs in inflammatory diseases [22]; the 5th and 95th percentiles of ALBU (3.0 to 4.7 g/dL) translated to a 34 to − 14.9% effect on linear CL and − 36 to 24% effect on *C*_min,ss_. Hypoalbuminemia is a prognostic factor in cancer patients and typically reflects low systemic levels of protein, as a consequence of cancer cachexia and/or inflammation. This is likely related to increased protein (including IgG) catabolism [23], which in parallel increases the clearance of trastuzumab. For example, abnormal ALBU values are commonly seen in patients with cachexia, and in this analysis it was more common for EBC patients than MBC patients to have elevated ALBU.

SGOT was not an unexpected covariate; SGPT was found to be a covariate in EBC to roughly the same extent and direction as the previous model in patients with EBC [4], and SGOT and SGPT are typically highly correlated. SGOT/SGPT may be correlated to hepatic inflammation due to LMET or previous treatment with chemotherapy, which in turn is correlated to increased clearance.

Inter-occasion variability was not estimated in this analysis. While the inter-subject variability reported is unaffected by lack of inter-occasion variability estimation, it is acknowledged that residual variability estimates might be inflated due to the day-to-day variability on PK [24]. Nevertheless, this would not affect the prediction of PK exposure, which is based on typical PK parameters, covariates, and inter-subject variabilities. As described by this model, trastuzumab PK exposure was lower in patients with gastric cancer than in patients with breast cancer. The difference in CL between the patients with MBC and AGC translated into 30.5% lower* C*_min,ss_ in the AGC population (Table 2). Similar trends were also found for *C*_max,ss_ and AUC_ss_. The physiological reasons for these observations are unknown and have also been reported previously [5, 25, 26].

The exploratory analysis of shed-antigen (ECD-HER2) suggested that patients with greater HER2-ECD levels had greater nonlinear clearance (lower *K*_m_). Since shed-antigen levels correlated to tumor size in nonclinical studies, shed-antigen levels may reflect target-mediated elimination of trastuzumab by HER2 receptors in systemic circulation. This hypothesis is supported by the work of Malik et al. [27], who concluded that the effect of ECD on trastuzumab CL can be fully attributed to the physical mechanism where trastuzumab binds to ECD and is then cleared as an immune complex. This relationship appeared to be confounded by the correlation of shed-antigen to SGOT levels.

No correlations with ECD-HER2 levels were observed for other explored covariates, including clinical outcome (relapse versus no relapse for EBC patients), neoadjuvant versus adjuvant for EBC patients or prior gastrectomy for AGC patients, gender, age, race (Asian versus non-Asian), hepatic function (SGPT and TBIL), renal function (CrCL), ALK, ECOG status, the number of metastatic sites, and HER2 expression level.

In two clinical trials of trastuzumab in MBC, where clinical efficacy and safety was observed, the mean trastuzumab *C*_min,ss_ following q3w dosing was > 50 µg/mL [28, 29]. This was consistent with data obtained from nonclinical xenograft models, which showed that tumor growth inhibition was maximum when trastuzumab concentrations were > 20 µg/mL [30–33; Roche data on file]. To maintain the efficacy of trastuzumab, it may be necessary to keep trastuzumab *C*_min_ similar to those observed in the previous clinical trials. Therefore, it is important to readminister the loading dose in the case of missed doses to minimize PK underexposure of trastuzumab and to quickly re-establish steady-state concentrations.

The recommendation for the readministration of a loading dose was evaluated based on the time required for *C*_min,ss_, after missing a dose, to return within 15% of the *C*_min,ss_ level given no missed dose. The criterion was set to 15%, as this was the same cutpoint applied to covariates in the analysis, i.e., eliminated covariates were to have less than a 15% effect on either *C*_min,ss_, *C*_max,ss_, or AUC_ss,_ and the 15% criterion has been proposed in the literature [34]. The 15% criterion used here is conservative and substantially smaller than the expected overall variability in trastuzumab PK.

The simulations supported the recommendation of readministering a loading dose (of 4 mg/kg or 8 mg/kg, depending on whether the patient is on the qw or q3w regimen, respectively), followed by the usual maintenance dose (of 2 mg/kg qw or 6 mg/kg q3w) in cases where a BC or AGC patient misses a dose by more than 1 week. In patients missing a dose by 1 week or less, the maintenance dose of 2 mg/kg qw or 6 mg/kg q3w should be administered without a reloading dose. This reloading and missed-dose regimen recommendation is reflected within the Herceptin^®^ prescribing information [35]. These guidelines are applicable regardless of whether the dose is missed in the early cycles (pre-steady state) or late cycles (steady state).

There are clinical scenarios where adequate trastuzumab washout from the systemic serum circulation is needed for safety such as pregnancy, breastfeeding, or administration of anthracycline-based therapy [36]. The simulations for the drug washout period indicate that at least 95% of patients with BC will reach concentrations that are < 1 µg/mL (approximately 3% of the *C*_min,ss_ of a typical patient with BC, or about 97% washout) by slightly longer than 6 months. Based on these results, conservatively a pregnancy washout period of 7 months is recommended. Despite the overall faster clearance and lower serum concentrations in patients with AGC, the same washout period of 7 months is also recommended in these patients, as some patients with AGC have similar serum concentrations as patients with BC. The Herceptin^®^ prescribing information reflects the 7-month serum washout period during which patients should avoid pregnancy, breastfeeding, or anthracycline-based therapy [35, 36].

In conclusion, a robust population PK model was developed for IV trastuzumab (Herceptin^®^) that adequately describes the serum concentration–time profile. This two-compartment model with parallel linear and nonlinear elimination is developed across various primary tumor types, disease status doses, and schedules. Patients with MBC and EBC had the same PK parameters (e.g., CL and *V*_c_), although patients with EBC had a higher steady-state exposure (*C*_min,ss_) due to differences in covariate distributions reflective of their localized disease. AGC was found to have a higher linear CL and *V*_c_ which translated into a 33% lower *C*_min,ss_ than patients with breast cancer. The most important PK covariates were WT, SGOT, ALBU and the presence of LMET. Given the overall variability in trastuzumab PK and the magnitude of these covariate effects, none of these patient factors requires any dose adjustment for the approved qw or q3w regimens. Simulations using the population PK model informed the prescribing information for Herceptin^®^; trastuzumab has a 7-month serum washout period during which patients should avoid an anthracycline-based therapy, pregnancy, or breastfeeding. A reloading dose is required if a maintenance dose is missed by > 1 week to maintain serum concentrations.

## Electronic supplementary material

Below is the link to the electronic supplementary material.

Supplementary material 1 (DOCX 3407 KB)

## References

[1] Kumar R, Yarmand-Bagheri R (2001). The role of HER2 in angiogenesis. Semin Oncol.

[2] Yakes FM, Chinratanalab W, Ritter CA, King W, Seelig S, Arteaga CL (2002). Herceptin-induced inhibition of phosphatidylinositol-3 kinase and Akt is required for antibody-mediated effects on p27, cyclin D1, and antitumor action. Cancer Res.

[3] Bruno R, Washington CB, Lu JF, Lieberman G, Banken L, Klein P (2005). Population pharmacokinetics of trastuzumab in patients with HER2 + metastatic breast cancer. Cancer Chemother Pharmacol.

[4] Quartino AL, Hillenbach C, Li J (2016). Population pharmacokinetic and exposure-response analysis for trastuzumab administered using a subcutaneous “manual syringe” injection or intravenously in women with HER2-positive early breast cancer. Cancer Chemother Pharmacol.

[5] Cosson VF, Ng VW, Lehle M, Lum BL (2014). Population pharmacokinetics and exposure-response analyses of trastuzumab in patients with advanced gastric or gastroesophageal junction cancer. Cancer Chemother Pharmacol.

[6] Keizer RJ, Huitema AD, Schellens JH, Beijnen JH (2010). Clinical pharmacokinetics of therapeutic monoclonal antibodies. Clin Pharmacokinet.

[7] Cobleigh MA, Vogel CL, Tripathy D (1999). Multinational study of the efficacy and safety of humanized anti-HER2 monoclonal antibody in women who have HER2-overexpressing metastatic breast cancer that has progressed after chemotherapy for metastatic disease. J Clin Oncol.

[8] US Food and Drug Administration (FDA) (1999) Guidance for industry: population pharmacokinetics. https://www.fda.gov/downloads/drugs/guidances/UCM072137.pdf. Accessed 13 Apr 2018

[9] European Medicines Agency (EMA); Committee for Medicinal Products for Human Use (CHMP) (2007) Guideline on reporting the results of population pharmacokinetic analyses. Doc. Ref. CHMP/EWP/185990/06. http://www.ema.europa.eu/docs/en_GB/document_library/Scientific_guideline/2009/09/WC500003067.pdf. Accessed 13 Apr 2018

[10] Bernadou G, Campone M, Merlin JL (2016). Influence of tumour burden on trastuzumab pharmacokinetics in HER2 positive non-metastatic breast cancer. Br J Clin Pharmacol.

[11] Gupta M, Lorusso PM, Wang B (2012). Clinical implications of pathophysiological and demographic covariates on the population pharmacokinetics of trastuzumab emtansine, a HER2-targeted antibody-drug conjugate, in patients with HER2-positive metastatic breast cancer. J Clin Pharmacol.

[12] Lu D, Girish S, Gao Y (2014). Population pharmacokinetics of trastuzumab emtansine (T-DM1), a HER2-targeted antibody–drug conjugate, in patients with HER2-positive metastatic breast cancer: clinical implications of the effect of covariates. Cancer Chemother Pharmacol.

[13] Ternant D, Azzopardi N, Raoul W (2018). Influence of antigen mass on the pharmacokinetics of therapeutic antibodies in humans. Clin Pharmacokinet.

[14] Ng CM, Joshi A, Dedrick RL, Garovoy MR, Bauer RJ (2005). Pharmacokinetic-pharmacodynamic-efficacy analysis of efalizumab in patients with moderate to severe psoriasis. Pharm Res.

[15] Dirks NL, Nolting A, Kovar A, Meibohm B (2008). Population pharmacokinetics of cetuximab in patients with squamous cell carcinoma of the head and neck. J Clin Pharmacol.

[16] Frey N, Grange S, Woodworth T (2010). Population pharmacokinetic analysis of tocilizumab in patients with rheumatoid arthritis. J Clin Pharmacol.

[17] Rosario M, Dirks NL, Gastonguay MR (2015). Population pharmacokinetics-pharmacodynamics of vedolizumab in patients with ulcerative colitis and Crohn’s disease. Aliment Pharmacol Ther.

[18] Ma P, Yang BB, Wang YM (2009). Population pharmacokinetic analysis of panitumumab in patients with advanced solid tumors. J Clin Pharmacol.

[19] Zhu Y, Hu C, Lu M (2009). Population pharmacokinetic modeling of ustekinumab, a human monoclonal antibody targeting IL-12/23p40, in patients with moderate to severe plaque psoriasis. J Clin Pharmacol.

[20] Xu ZH, Lee H, Vu T (2010). Population pharmacokinetics of golimumab in patients with ankylosing spondylitis: impact of body weight and immunogenicity. Int J Clin Pharmacol Ther.

[21] Chetty M (2018). Large molecules with large pharmacokinetic variability: progress in pursuit of key considerations for intersubject variability. Int J Pharmacokinet.

[22] Fasanmade AA, Adedokun OJ, Ford J (2009). Population pharmacokinetic analysis of infliximab in patients with ulcerative colitis. Eur J Clin Pharmacol.

[23] Ryman JT, Meibohm B (2017). Pharmacokinetics of monoclonal antibodies. CPT Pharmacometrics Syst Pharmacol.

[24] Karlsson MO, Sheiner LB (1993). The importance of modeling interoccasion variability in population pharmacokinetic analyses. J Pharmacokinet Biopharm.

[25] Han K, Jin J, Maia M, Lowe J, Sersch MA, Allison DE (2014). Lower exposure and faster clearance of bevacizumab in gastric cancer and the impact of patient variables: analysis of individual data from AVAGAST phase III trial. AAPS J.

[26] Kang YK, Rha SY, Tassone P (2014). A phase IIa dose-finding and safety study of first-line pertuzumab in combination with trastuzumab, capecitabine and cisplatin in patients with HER2-positive advanced gastric cancer. Br J Cancer.

[27] Malik PRV, Hamadeh A, Phipps C, Edginton AN (2017). Population PBPK modelling of trastuzumab: a framework for quantifying and predicting inter-individual variability. J Pharmacokinet Pharmacodyn.

[28] Leyland-Jones B, Gelmon K, Ayoub JP (2003). Pharmacokinetics, safety, and efficacy of trastuzumab administered every three weeks in combination with paclitaxel. J Clin Oncol.

[29] Baselga J, Carbonell X, Castañeda-Soto NJ (2005). Phase II study of efficacy, safety, and pharmacokinetics of trastuzumab monotherapy administered on a 3-weekly schedule. J Clin Oncol.

[30] Tunblad K, Lindbom L, McFadyen L (2008). The use of clinical irrelevance criteria in covariate model building with application to dofetilide pharmacokinetic data. J Pharmacokinet Pharmacodyn.

[31] Carter P, Presta L, Gorman CM (1992). Humanization of an anti-p185HER2 antibody for human cancer therapy. Proc Natl Acad Sci U S A.

[32] Pegram M, Hsu S, Lewis G (1999). Inhibitory effects of combinations of HER-2/neu antibody and chemotherapeutic agents used for treatment of human breast cancers. Oncogene.

[33] Pietras RJ, Fendly BM, Chazin VR (1993). Antibody to HER-2/neu receptor blocks DNA repair after cisplatin in human breast and ovarian cancer cells. Oncogene.

[34] Lewis GD, Figari I, Fendly B (1993). Differential responses of human tumor cell lines to anti-p185HER2 monoclonal antibodies. Cancer Immunol Immunother.

[35] US Food and Drug Administration (FDA) (2017) HERCEPTIN^®^ (trastuzumab) US Prescribing Information. https://www.accessdata.fda.gov/drugsatfda_docs/label/2017/103792s5337lbl.pdf. Accessed 13 Apr 2018

[36] European Medicines Agency (EMA) (2018) Herceptin summary of product characteristics. http://www.ema.europa.eu/docs/en_GB/document_library/EPAR_-_Product_Information/human/000278/WC500074922.pdf. Accessed 13 Apr 2018

