# Supplementary material for: Population pharmacokinetic and covariate analyses of intravenous trastuzumab (Herceptin®), a HER2-targeted monoclonal antibody, in patients with a variety of solid tumors
Source: Cancer Chemother Pharmacol. 2018 Nov 22;83(2):329–40. doi: 10.1007/s00280-018-3728-z (PMC6394489; doi:10.1007/s00280-018-3728-z)
Supplement: Supplementary file 1 — Supplementary material 1 (DOCX 3407 KB) [file 280_2018_3728_MOESM1_ESM.docx]

**Population pharmacokinetic and covariate analysis of intravenous trastuzumab (Herceptin^®^), a HER2-targeted monoclonal antibody, in patients with a variety of solid tumors**

**Authors:**

Angelica L. Quartino^1*^ · Hanbin Li^2^ · Whitney P. Kirschbrown^1^ · Ranvir Mangat^1,3^ · D. Russell Wada^2^ · Amit Garg^1^ ·
Jin Y. Jin^1^ · Bert Lum^1^

**Institutions:**

^1^Genentech, Inc., 1 DNA Way, South San Francisco, CA, 94080 USA

^2^Certara, L.P., 845 Oak Grove Ave, Menlo Park, CA, 94025 USA

^3^Current affiliation: Insight Rx, 233 Stanyan Street, San Francisco, CA, 94118 USA

*** Corresponding author:**

Angelica L. Quartino, PhD., 1 DNA Way, Mail Stop 463A, Genentech, Inc., South San Francisco, CA, 94080 USA

Phone: +1 650 303-1463; Fax: +1-650-467-5695; Email: [quartino.angelica@gene.com](mailto:quartino.angelica@gene.com)

**Online Resources**

**Online Resource 1.** List of studies included in the population PK analysis

| **Study** | **Phase** | **Primary tumor type** | **Dose/regimen/PK sampling** | **No. of patients  with PK** |
| --- | --- | --- | --- | --- |
| **H0407g** | I | Mainly  MBC | Single-agent, single-dose  IV: 10, 50, 100, 250, 500 mg  Infusion 90 min  PK: at pre-dose, 10 and 30 min, 2, 5, 10 and 24 h, and 2, 3, 7, 10, 14, 21, and 28 days after the end of infusion. | 16 |
| **H0551g** | II | MBC | Single-agent, multiple-dose  IV: 250 mg loading dose, then 100 mg qw for 10 weeks  Infusion 90 min  PK: pre-dose and 1 h after infusion up to Week 11. | 46 |
| **H0649g** | III | MBC | Single-agent, multiple-dose  IV: 4 mg/kg loading, then 2 mg/kg qw  Infusion 90 min, then 30 min  PK: pre-dose and at the end of infusion throughout the  treatment period. | 212 |
| **H0648g** | III | MBC | Trastuzumab + anthracycline + cyclophosphamide or paclitaxel chemotherapy, multiple-dose until progression  IV: 4 mg/kg loading, then 2 mg/kg qw  Infusion 90 min, then 30 min  PK: pre-dose and at the end of infusion throughout the  treatment period. | 219 |
| **M77004** | I | MBC | Trastuzumab + paclitaxel for 9 weeks, then Herceptin single agent until progression  IV: 4 mg/kg loading, then 2 mg/kg qw  Infusion 90 min  PK: multiple samples at Cycles 1–6, pre-dose at Cycles 7, 10, 13, 16, 18, and every 4 cycles thereafter | 16 |
| **BO15899** | II | NSCLC | Trastuzumab + gemcitabine  IV: 4 mg/kg loading, then 2 mg/kg qw until disease progression  Infusion 90 min then 30 min  PK: pre-dose, day 8 of Cycle 1, day 8 of Cycles 2–6, 8, and 14 | 51 |
| **BO15935** | I/II | MBC | Trastuzumab + paclitaxel  IV: 8 mg/kg loading, then 6 mg/kg q3w  Infusion: 90 min  PK: dense sample at Cycles 4 and 12 Trough and peak Cycles 1–13 | 32 |
| **WO16229** | II | MBC | Single-agent, multiple-dose  IV: 8 mg/kg loading, then 6 mg/kg q3w  Infusion: 90 min  PK: Peak and trough at each cycle for 98 patients  Full PK at Cycle 6 in 23 patients | 100 |
| **MO16982** | I//II | MBC | Single-agent, multiple-dose  IV: 6 mg/kg on Days 1, 8, and 15 as loading doses followed by 6 mg/kg q3w  Infusion: 90 min  PK: pre-dose, end of infusion and 1.5 h at Cycles 1, 2, 3, and 4. More samples at Cycles 2 and 4 (majority did not complete the planned PK sampling) | 72 |
| **BO22227/ HannaH** | III | EBC | Neoadjuvant with chemo (3 cycles of docetaxel and 3 cycles of FEC), adjuvant w/o chemo, multiple-dose  IV: 8 mg/kg loading, then 6 mg/kg q3w  SC: 600 mg fixed dose q3w  Infusion: 90 min then 30 min  PK: Pre-dose, end of infusion, Days 2, 15 at Cycles 1 and 9. Days 2, 4, 8, and 15 at Cycles 7 and 12. Pre-dose and end of infusion of each cycle | 298 in IV group |
| **BO18255/ToGA** | III | AGC | Trastuzumab + 5-FU and cisplatin, multi-dose  IV: 8 mg/kg loading, then 6 mg/kg q3w until disease progress  Infusion 90 min, then 30 min  PK: Cycle 1: pre-dose, end of infusion, Days 8 and 15. Cycle 2: pre-dose. Cycle 4: Pre-dose, and end of infusion. Cycle 6: Pre-dose | 266 |
| **BP22023/ CP2** | I | HV/EBC | Single-agent, single-dose  IV: 6 mg/kg  Infusion: 90 min  SC: 8 to 12 mg/kg PK: Day 1 pre-dose, 1.5 h, 3 h, Days 2, 3, 5, 6, 15, 22, 35, 43, 85 | 12 in IV group |
| **H0452g** | I | Mainly MBC | Single-agent, multiple-dose  IV: 10, 50, 100, 250, 500 mg, qw  Infusion: 90 min  PK: pre-dose, 1 h after infusion (main study period); weekly during maintenance. | 17 |
| **H0453g** | I | Mainly MBC | Trastuzumab + cisplatin, multiple-dose  IV: 10, 50, 100, 250, 500 mg, qw for 9 doses  Infusion 90 min  PK: pre-dose, 1 h after infusion on Days 0, 7, 14, 21, 28, 35, 42, 49, and 56 | 15 |
| **H0552g** | II | MBC | Trastuzumab + cisplatin, multiple-dose  IV: 4 mg/kg loading, then 2 mg/kg qw for 8 weeks  Infusion: 90 min  PK: pre-dose, 1 h after infusion on Days 0, 7, 14, 21, 28, 35, 42, 49, and 56 | 37 |
| **H1995** | I | MBC | Trastuzumab + doxorubicin and cyclophosphamide  IV: 4 mg/kg loading, then 2 mg/kg qw  Infusion: 90 min then 30 min  PK: pre-dose, end of infusion at Day 1 and Weeks 2–8, every 4 weeks thereafter. | 17 |
| **BO16348/HERA** | III | EBC | Single-agent, multiple doses  IV: 8 mg/kg loading, then 6 mg/kg q3w for 1 year or 2 years  Infusion: 90 min for all doses  PK: Rich sampling Cycle 1, 13, 18, 35; pre-dose, 1.5, 2, 3, 4, 6, 8, 24 h, and 7, 14, 28, 42 days post-dose | 93 |
| **H4613g** | Ib (DDI) | HER2-positive solid malignancy | Trastuzumab + docetaxel and carboplatin, multiple-dose  IV: 6 mg/kg on Day 2 and Day 8 of Cycle 1, then Day 1 of each subsequent cycle (21 days per cycle)  Infusion: 90 min  PK: pre-dose, Days 2 and 8 at Cycle 1; Day 1 at cycles 2 and 3 | 56 |

*DDI* drug–drug interaction, *FEC* fluorouracil, epirubicin, and cyclophosphamide, *5-FU* 5-fluorouracil, *IV* intravenous, *MBC* metastatic breast cancer, *PK* pharmacokinetic, *qw* weekly, *q3w* every 3 weeks

**Online Resource 2.** Handling of outliers

Pharmacokinetic samples were identified as outliers by comparing trastuzumab concentrations before and after dosing. Typically, outliers occurred when the minimum serum concentration exceeded peak concentrations (*n* = 510 samples). Outliers were also identified using an initial two-compartment model with linear and nonlinear elimination from the central compartment, inter-individual variability on clearance, central volume, and peripheral volume, and proportional plus additive residual error model (*n* = 799 samples). Data with weighted residuals > 3 or conditional weighted residuals > 3 or individual weighted residuals > 3 were considered as potential outliers which were confirmed by examining the concentration versus time profiles. Finally, two patients with an unusual pharmacokinetic profile were removed (*n* = 21 samples) (Online Resource 3). A sensitivity analysis on the final model was done to compare estimated pharmacokinetic parameters with and without those outliers.

**Online Resource 3.** Overview of data exclusions for PopPK model development

| Category | Description | Patients | Samples (%) |
| --- | --- | --- | --- |
| Valid samples | Including samples from the treatment phase of studies and above BQL* | 1588 | 27426 |
| Data exclusions | Missing dose information. PK records collected after the missing dose were excluded | 9 (1^†^) | 56 (0.2%) |
|  | Trough concentration greater than peak concentration | 182 | 510 (1.9%) |
|  | \|WRES\|>3 or \|CWRES\|>3 or \|IWRES\|>3 outliers | 386 (3^†^) | 799 (2.9%) |
|  | Unusual PK profile (IDs 7 and 9 in study H0407g) | 2 (2^†^) | 21 (0.08%) |
| Analyzed samples | Used for model development | 1582 | 26040 (94.9%) |

* Samples from the follow-up phase of studies had no associated dose information and were not considered valid (*n* = 1,978, PLDS = 1 in the NONMEM dataset). Also PK samples below BQL (*n* = 316, BQL = 1 in the NONMEM dataset) were excluded

^†^ Numbers of patients completely excluded are indicated in parentheses

*BQL* below the limit of quantification, *CWRES* conditional weighted residual(s), *IWRES* individual weighted residual(s), *PK* pharmacokinetic, *WRES* weighted residual(s)

**Online Resource 4.** Model evaluation

Model evaluation was performed by goodness-of-fit plots, nonparametric bootstrap resampling (*n* = 200) techniques stratified by study and dose group, and visual and numerical predictive checks (*n* = 1000 trial replicates) stratified by primary tumor type and dose regimen. The extent of shrinkage of estimation derived from the final model was assessed for each inter-individual variability term (*η*) as well as for residual error (*ε*) [Savic RM, Karlsson MO (2009) Importance of shrinkage in empirical Bayes estimates for diagnostics: problems and solutions. AAPS J 11:558–569].

**Online resource 5.** three-step forward addition and backward elimination approach was used to identify covariates

A three-step forward addition and a three-step backward elimination approach were used for identification of the covariates. In the first forward step, baseline WT was incorporated on linear clearance, central volume, and peripheral volume because it was shown to have significant impact in previous analyses, before screening other covariates. The second forward step consisted of a stepwise univariate screening of all covariates followed by inclusion of the covariates with the largest change in objective function. In the third forward step, univariate screening of all other covariates was performed and significant covariates (*p* < 0.005) were incorporated simultaneously into a full model. In the first stepwise backward elimination step, each covariate was removed one at a time, and the one with the least impact on the objective function removed. This process was repeated until all remaining covariates were statistically significant (*p* < 0.001). Next, a second backward elimination step was performed for primary tumor type where each patient population was compared to the metastatic breast cancer (MBC) reference population. Finally, additional covariates were eliminated using a clinical relevance criterion that was set to 15%, i.e., covariates were eliminated if they had < 15% effect on steady-state minimum serum concentration (C_min_), maximum serum concentration (C_max_), and area under the curve (AUC). Other covariates of interest, including the neoadjuvant versus adjuvant treatment for early breast cancer (EBC), prior gastrectomy in advanced gastric cancer, shed-antigen level in MBC and non-small cell lung cancer, and clinical outcome (relapse versus no relapse) in EBC, which were only available for a portion of the population, were not included in the covariate selection process. Instead, exploratory analyses were conducted where the covariates were tested one at the time using the final statistical covariate pharmacokinetic (PK) model to estimate the statistical significance (*p* < 0.001). The impact on PK was also evaluated by graphically examining the correlation between each covariate and the Empirical Bayes Estimates of C_min_, C_max_, and AUC.

**Online Resource 6.** Summary of PK data and patient characteristics

| **Data overview** | **Value**  **Median (5^th^–95^th^ percentile)** | |
| --- | --- | --- |
| Number of patients | 1,588 | |
| Number PK samples | 27,426 | |
| Number of dose per patient | 11 (2 to 43) | |
| Number of PK samples per patient | 13 (2 to 38) | |
| **Continuous variables** | **Unit** | **Median (5^th^–95^th^ percentile)** |
| Age | yr | 53 (33 to 72) |
| Baseline Weight | kg | 66 (48 to 95) |
| Baseline ALBU | L/dL | 4 (3 to 4.7) |
| Baseline ALKP | U/L | 99 (47 to 373) |
| Baseline TBIL | mg/dL | 0.5 (0.2 to 1.1) |
| Baseline SGPT | IU/L | 22 (9 to 80) |
| Baseline SGOT | IU/L | 24 (14 to 103) |
| Baseline CrCL | mL/min | 90 (54 to 158) |
| Baseline SHED (*n* = 717 MBC, NSCLC) | ng/mL | 15 (1.7 to 658) |
| **Categorical variables** | **Category** | **N (%)** |
| Primary tumor type or healthy volunteer subject | MBC  EBC  AGC  NSCLC and other types  HV | 810 (51.0)  391 (24.6)  274 (17.3)  107 (6.7)  6 (0.4) |
| Sex | Male  Female | 274 (17.3)  1314 (82.7) |
| Race | Asian  Non-Asian | 264 (16.6)  1324 (83.4) |
| HER2 over-expression level | IHC1+ or IHC2+  IHC3+  Unknown | 412 (25.9)  1087 (68.5)  89 (5.6) |
| Baseline ECOG score | 0–1  ≥2  Unknown | 1500 (94.5)  61 (3.8)  27 (1.7) |
| Baseline number of metastatic sites | 0-3  >3  Unknown | 1289 (81.2)  197 (12.4)  102 (6.4) |
| Baseline liver metastases | Yes  No  Unknown | 497 (31.3)  1031 (64.9)  60 (3.8) |
| Prior gastrectomy (AGC patients only) | Yes  No  Unknown | 200 (12.6)  70 (4.4)  4 (0.3) |
| EBC treatment phase (time varying)* | Neoadjuvant  Adjuvant | 298 (18.1)  369 (22.4) |
| Dosing regimen | single dose  weekly  3-weekly | 28 (1.8)  643 (40.5)  917 (57.7) |
| Chemotherapy (time varying)* | Single-agent  Anthracycline combinations  Docetaxel combinations  Paclitaxel combinations  Cisplatin combinations  Other chemotherapies | 1188 (72.2)  458 (27.8)  355 (21.6)  151 (9.2)  373 (22.7)  22 (1.3) |
| Clinical Outcome  (BO16348/HERA patients only) | Relapse  No-relapse | 23 (0.9)  70 (2.7) |
| * Note: The same EBC patient could have received treatment during both the neoadjuvant and the adjuvant phase. The same goes for chemotherapy, where the same patient might have received trastuzumab as both single-agent and in combination with chemotherapy or with different types of chemotherapy. So, number of patients for these two categories will not match the total number of patients in studies  *AGC* advanced gastric cancer, *ALBU* albumin, *ALKP* alkaline phosphatase, *CrCL* creatinine clearance, *EBC* early breast cancer, *ECOG* Easter Cooperative Oncology Group, *HV* healthy volunteer(s), *NSCLC* non-small cell lung cancer, *SGOT* aspartate aminotransferase, *SGPT* alanine aminotransferase, *SHED* shed=antigen ECD-HER2, *TBIL* total serum bilirubin | | |

**Online Resource 7.** Final model goodness-of-fit plots
Created using S-Plus Software package (Version 8.2, SolutionMetrics, Sydney, NSW, Australia)

Top panel: Observed versus individual predicted concentrations (left: log-scale; middle: normal scale) and CWRES versus time (right) for the final PK model. Bottom panel: Observed versus population predicted concentrations (left: log-scale; middle: normal scale), and CWRES versus population predicted concentrations (right) for the final PK model. Dashed lines are smooth (lowess) curves of the data

*CWRES* conditional weighted residual(s), *PK* pharmacokinetic

**Online Resource 8.** Impact of ALBU on model-predicted steady-state C_min,ss_ stratified by primary tumor type for an 8 mg/kg loading dose followed by 6 mg/kg q3w
Created using R Software package (Version 3.0, http://www.r-project.org/)


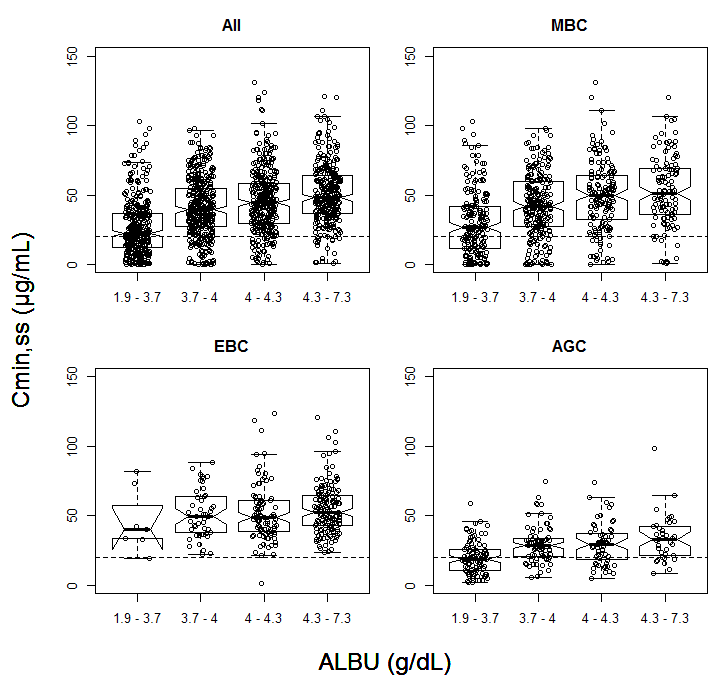


The PK variables were calculated using EBEs. The PK parameters were grouped by quartiles of baseline ALBU distribution. The box shows the median, 25^th^, and 75^th^ percentile of the patients in the group. The horizontal line is the 20 μg/mL targeted threshold concentration for efficacy identified in preclinical xenograft models

*ALBU* albumin, *EBEs* Empirical Bayes Estimates, *PK* pharmacokinetic

**Online Resource 9.** Impact of SGOT on model-predicted steady-state C_min,ss_ stratified by primary tumor types for an 8 mg/kg loading dose followed by 6 mg/kg q3w
Created using R Software package (Version 3.0, http://www.r-project.org/)


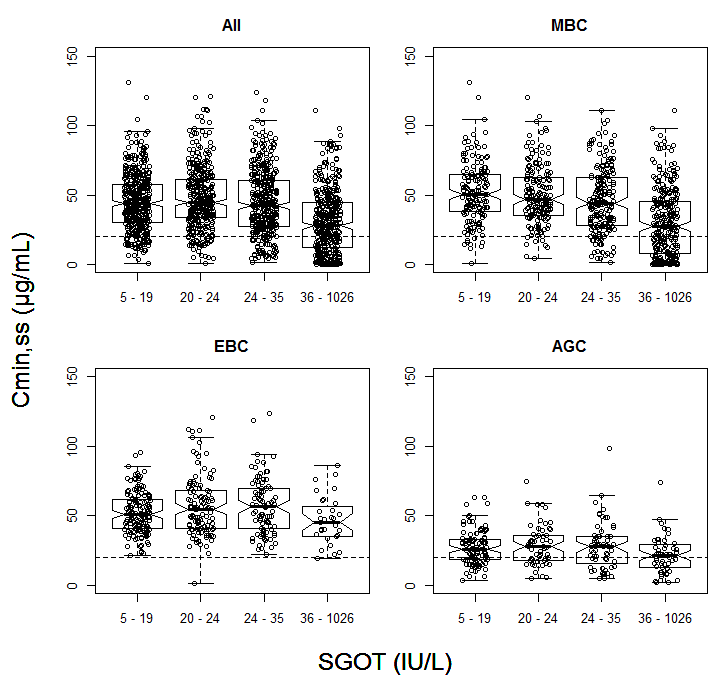


The PK variables were calculated using EBEs. The PK parameters were grouped by quartiles of baseline SGOT distribution. The box shows the median, 25^th^, and 75^th^ percentile of the patients per SGOT group. The horizontal line is the 20 μg/mL targeted threshold concentration for efficacy identified in preclinical xenograft models

*EBEs* Empirical Bayes Estimate(s), *SGOT* aspartate aminotransferase, *q3w* every 3 weeks

**Online Resource 10.** Sensitivity plot comparing the effect of covariates on the model-predicted exposure measures (C_min,ss,_ C_max,ss,_ and AUC_ss_) for an 8 mg/kg loading dose followed by 6 mg/kg q3w in a typical MBC patient
Created using R Software package (Version 3.0, http://www.r-project.org/)


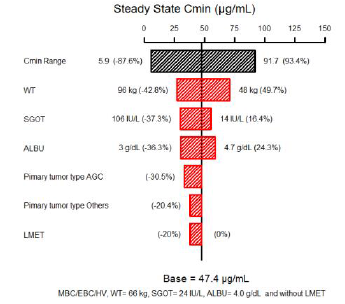

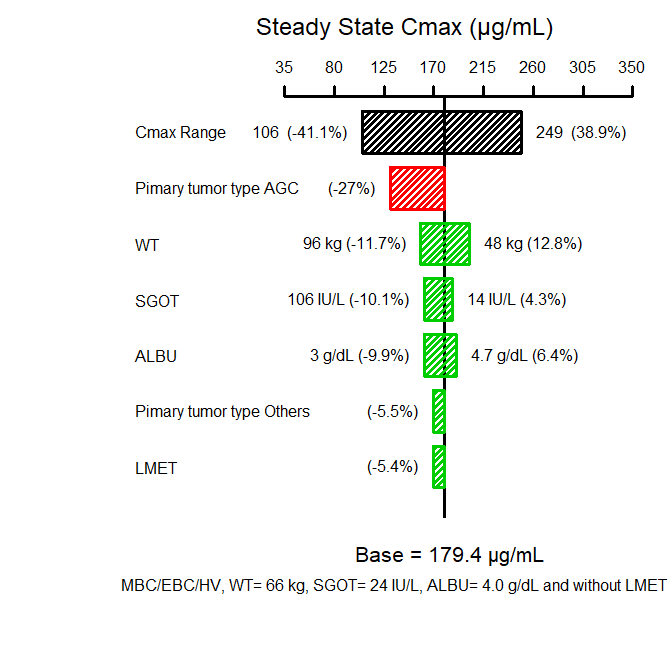

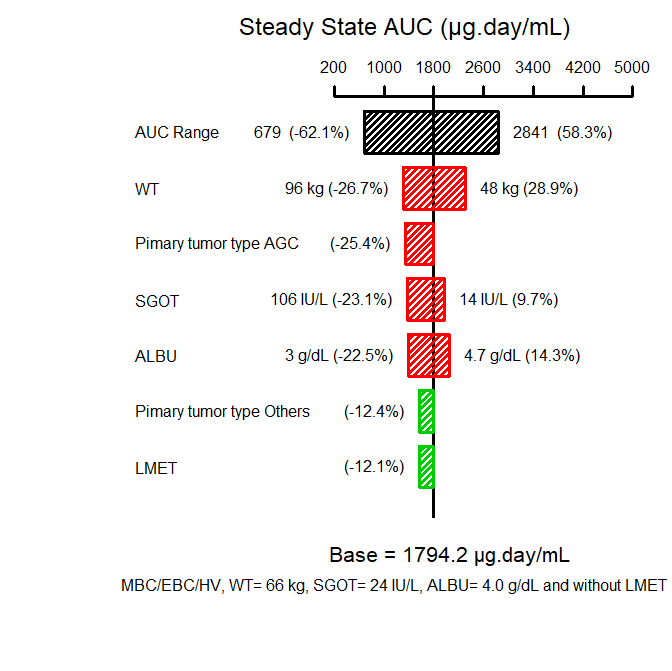


Note: C_min,ss_, C_max,ss,_ and AUC_ss_ bars describe the 5^th^ to 95^th^ percentile range in parameter value across the studied subjects. The WT, SGOT, and ALBU bars describe the range as the indicated covariate varies across its respective 5^th^ to 95^th^ percentile range. The vertical line is a reference parameter value in a typical MBC patient with median covariate values. Population ranges were simulated using *post hoc* PK parameters normalized to a MBC patient with WT = 66 kg. Red color bars have at least 15% changes in exposure, while green bars are less than 15%

*ALBU* albumin, *AUC_ss_* area under the curve at steady-state, *C_max,ss_* maximum steady-state serum concentration, *C_min,ss_* minimum steady-state serum concentration, *MBC* metastatic breast cancer, *PK* pharmacokinetic, *SGOT* aspartate aminotransferase, *q3w* every 3 weeks, *WT* body weight

**Online Resource 11.** Model-predicted steady-state C_min,ss_ versus SHED for an 8 mg/kg loading dose followed by 6 mg/kg q3w, using the final model
Created using R Software package (Version 3.0, http://www.r-project.org/)


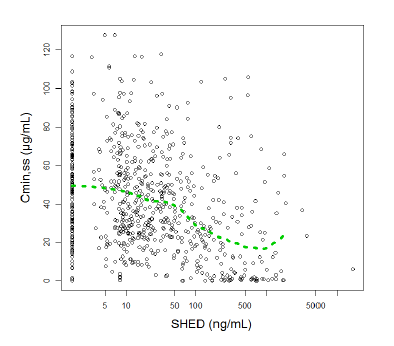


Note: Circles are PK variables calculated using EBEs. The green line shows a smooth trend (lowess)

*C_min,ss_ minimum steady-state serum concentration, EBEs* Empirical Bayes Estimate(s), *PK* pharmacokinetic, *q3w* every 3 weeks, *SHED* shed-antigen ECD-HER2

**Online Resource 12.** Linear, nonlinear, and total clearance versus trastuzumab serum concentration for MBC/EBC (left) and AGC (right) patients

Created using R Software package (Version 3.0, http://www.r-project.org/)


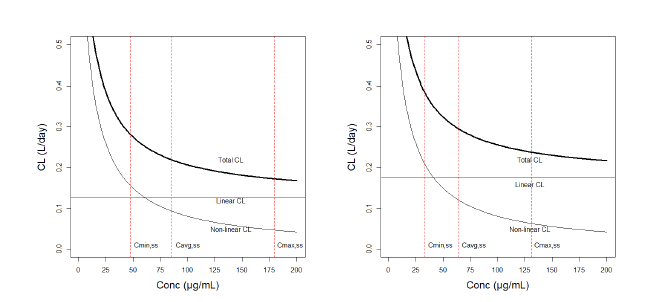


Vertical lines indicate C_max,ss_, C_min,ss_, and C_avg,ss_ at steady state (Cycle 7) for an 8 mg/kg IV

loading dose followed by 6 mg/kg q3w for patients WT = 66 kg, SGOT = 24 IU/L, ALBU = 4.0 g/dL, and

without LMET

*AGC* advanced gastric cancer, *ALBU* albumin, *C_avg,ss_* average steady-state serum concentration, *C_min,ss_* minimum steady-state serum concentration, *EBC* early breast cancer, *IV* intravenous, *LMET* liver metastases, *MBC* metastatic breast cancer, *SGOT* aspartate aminotransferase, *q3w* every 3 weeks

**Online Resource 13.** Simulated trastuzumab serum concentrations following the 4 mg/kg loading dose + 2 mg/kg qw dosing regimen with no delays in dosing

Created using R Software package (Version 3.0, http://www.r-project.org/)


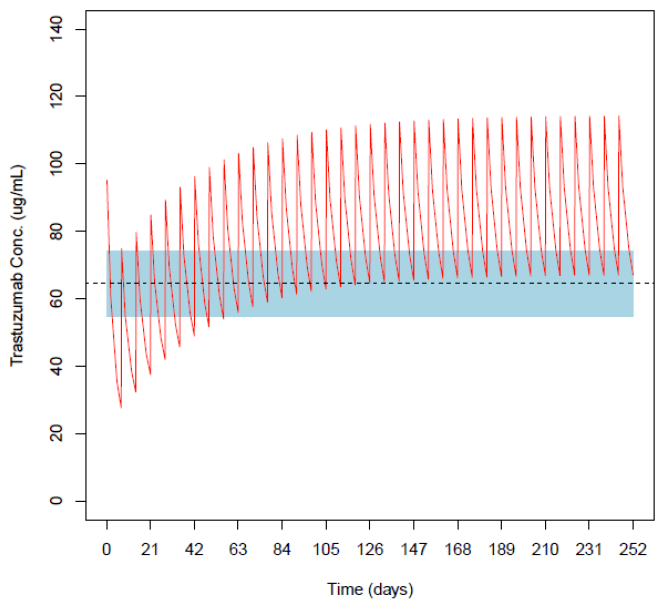


The red line represents a typical patient with BC where WT = 66 kg, SGOT = 24 IU/L, ALBU = 4 g/dL, and is without LMET. The black dashed line and blue shaded area represents the C_min,ss_ levels when there is no missed dosing (64.5 μg/mL, at pre-dose Cycle 19) and 15% range, respectively

*ALBU* albumin, *BC* breast cancer, *C_min,ss_* minimum steady-state serum concentration, *LMET* liver metastases, *qw* weekly, *SGOT* aspartate aminotransferase, *WT* body weight

**Online Resource 14.** Simulated trastuzumab serum concentrations following the 8 mg/kg loading dose + 6 mg/kg q3w dosing regimen with no delays in dosing

Created using R Software package (Version 3.0, http://www.r-project.org/)
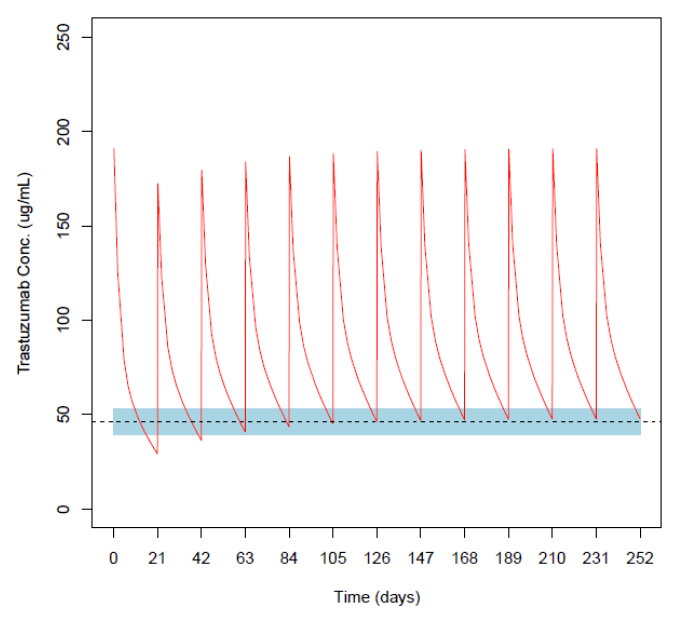


The red line represents a typical patient with BC where WT = 66 kg, SGOT = 24 IU/L, ALBU = 4 g/dL, and is without LMET. The black dashed line and blue shaded area represents the C_min,ss_ levels when there is no missed dosing (46.2 μg/mL, at pre-dose Cycle 7) and 15% range, respectively

*ALBU* albumin, *BC* breast cancer, *C_min,ss_* minimum steady-state serum concentration, *LMET* liver metastases, *q3w* every 3 weeks, *SGOT* aspartate aminotransferase, *WT* body weight

**Online Resource 15.** Simulated trastuzumab serum concentrations following the 4 mg/kg loading dose + 2 mg/kg qw dosing regimen with a 1-week delay in dosing where dosing is restarted with the 2 mg/kg maintenance dose

Created using R Software package (Version 3.0, http://www.r-project.org/)


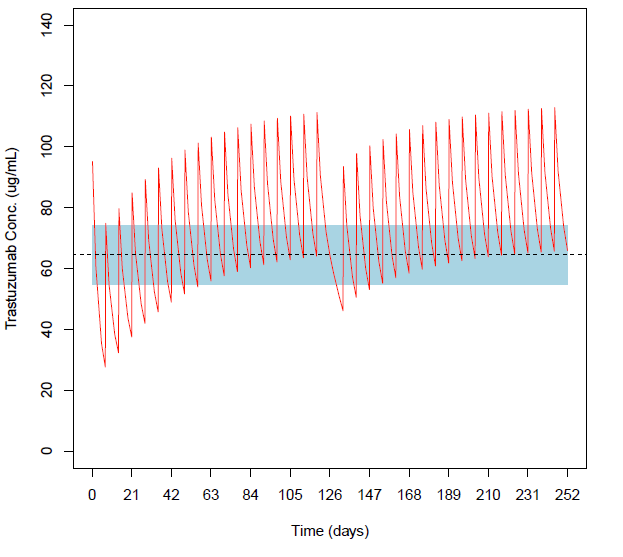


The red line represents a typical patient with BC where WT = 66 kg, SGOT = 24 IU/L, ALBU = 4 g/dL, and is without LMET. The black dashed line and blue shaded area represents the C_min,ss_ levels when there is no missed dosing (64.5 μg/mL, at pre-dose Cycle 19) and 15% range, respectively

*ALBU* albumin, *BC* breast cancer, *C_min,ss_* minimum steady-state serum concentration, *LMET* liver metastases, *qw* weekly, *SGOT* aspartate aminotransferase, *WT* body weight

**Online Resource 16.** Simulated trastuzumab serum concentrations following the 8 mg/kg loading dose + 6 mg/kg q3w dosing regimen with a 1-week delay in dosing where dosing is restarted with the 6 mg/kg maintenance dose

Created using R Software package (Version 3.0, http://www.r-project.org/)


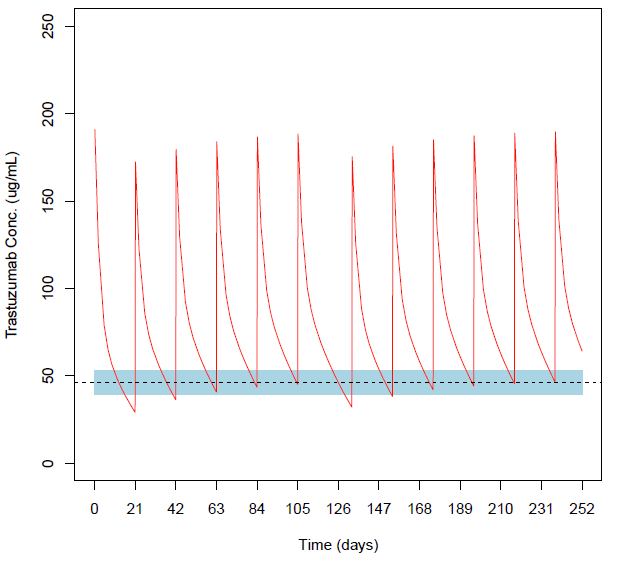


The red line represents a typical patient with BC where WT = 66 kg, SGOT = 24 IU/L, ALBU = 4 g/dL, and is without LMET. The black dashed line and blue shaded area represents the C_min,ss_ levels when there is no missed dosing (46.2 μg/mL, at pre-dose Cycle 7) and 15% range, respectively

*ALBU* albumin, *BC* breast cancer, *C_min,ss_* minimum steady-state serum concentration, *LMET* liver metastases, *q3w* every 3 weeks, *SGOT* aspartate aminotransferase, *WT* body weight

**Online Resource 17.** Simulated trastuzumab serum concentrations following the 4 mg/kg loading dose + 2 mg/kg qw dosing regimen with a 2-week delay in dosing where dosing is restarted with the 2 mg/kg maintenance dose

Created using R Software package (Version 3.0, http://www.r-project.org/)


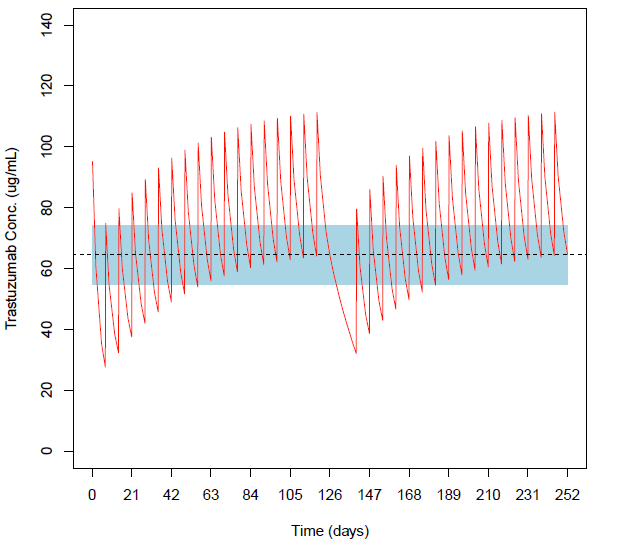


The red line represents a typical patient with BC where WT = 66 kg, SGOT = 24 IU/L, ALBU = 4 g/dL, and is without LMET. The black dashed line and blue shaded area represents the C_min,ss_ levels when there is no missed dosing (64.5 μg/mL, at pre-dose Cycle 19) and 15% range, respectively

*ALBU* albumin, *BC* breast cancer, *C_min,ss_* minimum steady-state serum concentration, *LMET* liver metastases, *qw* weekly, *SGOT* aspartate aminotransferase, *WT* body weight

**Online Resource 18.** Simulated trastuzumab serum concentrations following the 8 mg/kg loading dose + 6 mg/kg q3w dosing regimen with a 2-week delay in dosing where dosing is restarted with the 6 mg/kg maintenance dose

Created using R Software package (Version 3.0, http://www.r-project.org/)


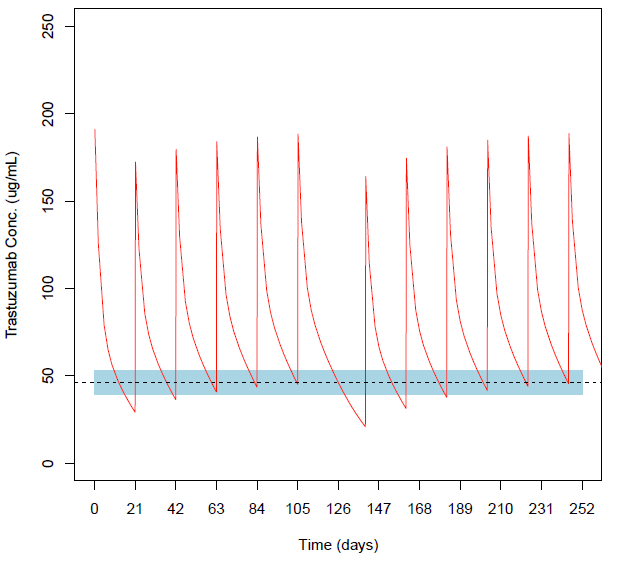


The red line represents a typical patient with BC where WT = 66 kg, SGOT = 24 IU/L, ALBU = 4 g/dL, and is without LMET. The black dashed line and blue shaded area represents the C_min,ss_ levels when there is no missed dosing (46.2 μg/mL, at pre-dose Cycle 7) and 15% range, respectively

*ALBU* albumin, *BC* breast cancer, *C_min,ss_* minimum steady-state serum concentration, *LMET* liver metastases, *q3w* every 3

weeks, *SGOT* aspartate aminotransferase, *WT* body weight

**Online Resource 19.** Simulated trastuzumab serum concentrations following the 4 mg/kg loading dose + 2 mg/kg qw dosing regimen with a 2-week delay in dosing where dosing is restarted with the 4 mg/kg loading dose followed by the 2 mg/kg maintenance dose

Created using R Software package (Version 3.0, http://www.r-project.org/)


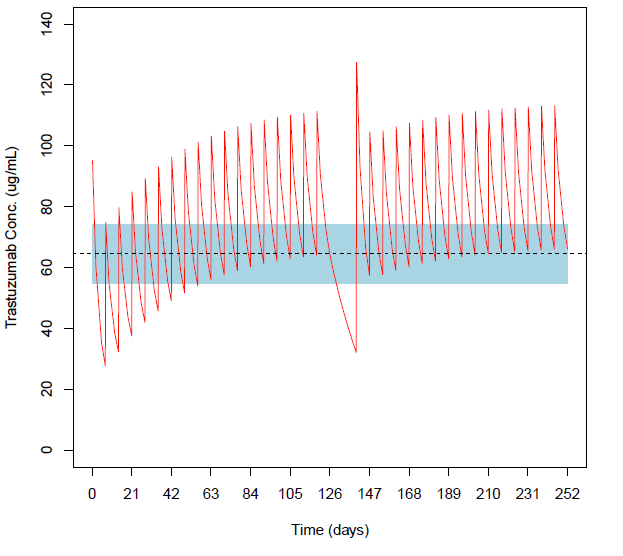


The red line represents a typical patient with BC where WT = 66 kg, SGOT = 24 IU/L, ALBU = 4 g/dL, and is without LMET. The black dashed line and blue shaded area represents the C_min,ss_ levels when there is no missed dosing (64.5 μg/mL, at pre-dose Cycle 19) and 15% range, respectively

*ALBU* albumin, *BC* breast cancer, *C_min,ss_* minimum steady-state serum concentration, *LMET* liver metastases, *qw* weekly, *SGOT* aspartate aminotransferase, *WT* body weight

**Online Resource 20.** Simulated trastuzumab serum concentrations following the 8 mg/kg loading dose + 6 mg/kg q3w dosing regimen with a 2-week delay in dosing where dosing is restarted with the 8 mg/kg loading dose followed by the 6 mg/kg maintenance dose

Created using R Software package (Version 3.0, http://www.r-project.org/)


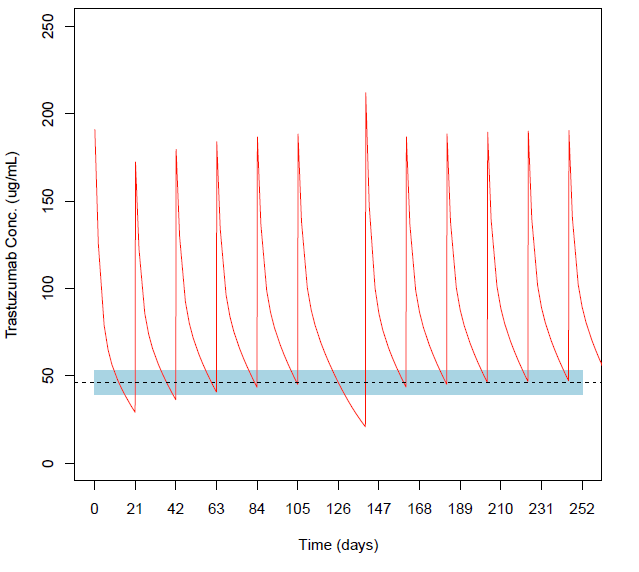


The red line represents a typical patient with BC where WT = 66 kg, SGOT = 24 IU/L, ALBU = 4 g/dL, and is without LMET. The black dashed line and blue shaded area represents the C_min,ss_ levels when there is no missed dosing (46.2 μg/mL, at pre-dose Cycle 7) and 15% range, respectively

*ALBU* albumin, *BC* breast cancer, *C_min,ss_* minimum steady-state serum concentration, *LMET* liver metastases, *q3w* every 3

weeks, *SGOT* aspartate aminotransferase, *WT* body weight
